# Jail-to-community treatment continuum for adults with co-occurring substance use and mental disorders: study protocol for a pilot randomized controlled trial

**DOI:** 10.1186/s13063-017-2088-z

**Published:** 2017-08-04

**Authors:** Richard A. Van Dorn, Sarah L. Desmarais, Candalyn B. Rade, Elizabeth N. Burris, Gary S. Cuddeback, Kiersten L. Johnson, Stephen J. Tueller, Megan L. Comfort, Kim T. Mueser

**Affiliations:** 1 Urban Health Program, RTI International, Research Triangle Park, 3040 E. Cornwallis Road, P.O. Box 12194, Durham, NC 27709 USA; 2 Department of Psychology, North Carolina State University, Raleigh, NC 27695 USA; 3 School of Social Work, University of North Carolina at Chapel Hill, Chapel Hill, NC 27599 USA; 4 Risk Behavior and Family Research Program, RTI International, Research Triangle Park, Durham, NC 27709 USA; 5 Center for Psychiatric Rehabilitation, Boston University, Boston, MA 02215 USA

**Keywords:** Jail, Mental illness, Substance use, Open trial, Randomized controlled trial

## Abstract

**Background:**

Adults with co-occurring mental and substance use disorders (CODs) are overrepresented in jails. In-custody barriers to treatment, including a lack of evidence-based treatment options and the often short periods of incarceration, and limited communication between jails and community-based treatment agencies that can hinder immediate enrollment into community care once released have contributed to a cycle of limited treatment engagement, unaddressed criminogenic risks, and (re)arrest among this vulnerable and high-risk population. This paper describes a study that will develop research and communication protocols and adapt two evidence-based treatments, dual-diagnosis motivational interviewing (DDMI) and integrated group therapy (IGT), for delivery to adults with CODs across a jail-to-community treatment continuum.

**Methods/design:**

Adaptations to DDMI and IGT were guided by the Risk-Need-Responsivity model and the National Institute of Corrections’ implementation competencies; the development of the implementation framework and communication protocols were guided by the Evidence-Based Interagency Implementation Model for community corrections and the Inter-organizational Relationship model, respectively. Implementation and evaluation of the protocols and adapted interventions will occur via an open trial and a pilot randomized trial. The clinical intervention consists of two in-jail DDMI sessions and 12 in-community IGT sessions. Twelve adults with CODs and four clinicians will participate in the open trial to evaluate the acceptability and feasibility of, and fidelity to, the interventions and research and communication protocols. The pilot controlled trial will be conducted with 60 inmates who will be randomized to either DDMI-IGT or treatment as usual. A baseline assessment will be conducted in jail, and four community-based assessments will be conducted during a 6-month follow-up period. Implementation, clinical, public health, and treatment preference outcomes will be evaluated.

**Discussion:**

Findings have the potential to improve both jail- and community-based treatment services for adults with CODs as well as inform methods for conducting rigorous pilot implementation and evaluation research in correctional settings and as inmates re-enter the community. Findings will contribute to a growing area of work focused on interrupting the cycle of limited treatment engagement, unaddressed criminogenic risks, and (re)arrest among adults with CODs.

**Trial registration:**

ClinicalTrials.gov, NCT02214667. Registered on 10 August 2014.

**Electronic supplementary material:**

The online version of this article (doi:10.1186/s13063-017-2088-z) contains supplementary material, which is available to authorized users.

## Background

More than two million adults with serious mental illnesses (SMIs), including schizophrenia-spectrum, bipolar, or major depressive disorders, are admitted to US jails annually [1]. A majority of justice-involved adults with SMI also have alcohol or drug use problems. Substance use among adults with SMI is a complicating treatment factor, as drug and alcohol use worsens illness trajectories [2–4], increases the cost of treatment [5], and is associated with multiple negative outcomes, including homelessness, arrest, and violence [6–8]. However, there are many jail-based barriers to treatment for co-occurring mental and substance use disorders (CODs), including a lack of evidence-based treatment options and the often short periods of incarceration. There also is limited communication between jails and community-based treatment agencies, which decreases the likelihood of immediate entry into community care upon release. As a result, justice-involved adults with CODs experience high rates of treatment failure, and jail-based and community-based services struggle to intervene effectively. Together, these issues have contributed to a cycle of limited treatment engagement, unaddressed criminogenic risks, and (re)arrest among this vulnerable and high-risk population [9–11].

Although evidence-based programs for CODs are available for inpatient and outpatient settings [2, 5, 10, 12, 13], effective treatment programs for justice-involved adults with CODs are limited [4, 6, 14–16]. Despite an inmate’s constitutional right to adequate healthcare [17], behavioral health treatment in jail is rarely evidence-based or focused on key leverage points, such as community re-entry or criminogenic risks. Several factors have contributed to this lack of evidence-based care, including jails’ limited capacity to respond to inmates’ behavioral health needs, the long-standing mission of incapacitation and punishment rather than rehabilitation, and the often short periods of incarceration and frequently unknown release dates for inmates, which limits the opportunities for and duration of in-jail treatment when available. Also, frequent and ongoing coordination with community-based agencies regarding inmates’ immediate enrollment in community services upon release from jail remains a challenge [18].

The issues are as follows: (1) Can existing evidence-based practices be adapted and delivered to justice-involved adults with CODs across both jail and community settings in a way that mitigates these barriers while also reducing re-arrest, substance use, and psychiatric symptoms and improving quality of life? (2) Can communication protocols be developed that facilitate immediate entry into community-based services upon release from jail? If so, what are the available and effective interventions that are portable to the jail setting and amenable to the inclusion of criminogenic risks? In addition to existing guidelines and recommendations for jail-based services [18, 19], the mental health services literature describes several strategies that focus on organizational and clinical changes and the development of effective intra- and inter-agency communication that improves outcomes associated with transitions from institutional to community care [20–24]. These include discharge planning, information sharing, monitoring of clients post-release, immediate and intensive community-based care, and peer support workers who span both the institutional and community settings. Still, the development and adoption of and adherence to guidelines, recommendations, and improvement interventions for clinical care with justice-involved adults with CODs have proven difficult in both the jail setting and during the transition to the community.

### Objectives

The objectives of the current study are to (1) develop research protocols to obtain access to a jail population, implement a rigorous research design, including randomization to a pilot trial, and ensure that participants do not perceive any undue coercion to participate; (2) develop communication protocols so that jail and community treatment are coordinated and linked for effective community re-entry; and (3) adapt the clinical content of extant evidence-based programs to better address both behavioral health and criminogenic needs of jail inmates with CODs, including anger management, harm reduction, illness management, criminal thinking, antisocial peer networks, and treatment motivation. The following research aims were designed to address these objectives:

- **Aim 1:** To adapt evidence-based interventions for delivery to jail inmates as they transition from institutional to community living, examining the initial acceptability of the adaptations through an uncontrolled open trial
- **Aim 2:** To conduct a pilot randomized controlled trial (RCT) to establish knowledge in three priority areas:
  - *Aim 2.1*: The feasibility and acceptability of the adapted interventions
  - *Aim 2.2*: The feasibility and acceptability of the proposed research and communication protocols
  - *Aim 2.3*: The effectiveness of and estimated effect sizes associated with the adapted interventions vis-à-vis reductions in re-arrest, substance use, and psychiatric symptoms, and improvements in uptake of usual care.

## Methods/design

**Fig. 1:**
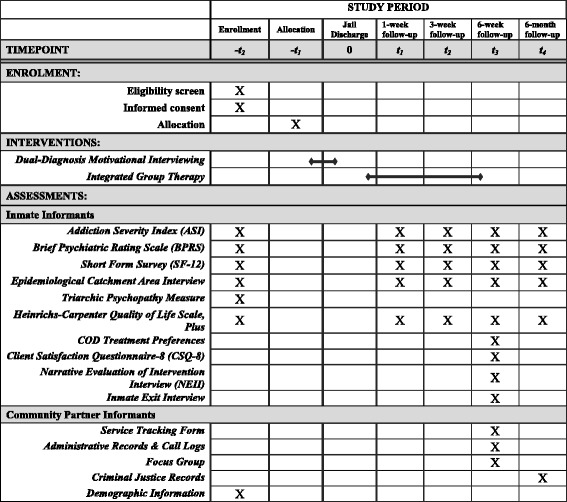
Pilot RCT enrollment, interventions, and assessments according to SPIRIT guidelines

**Fig. 2:**
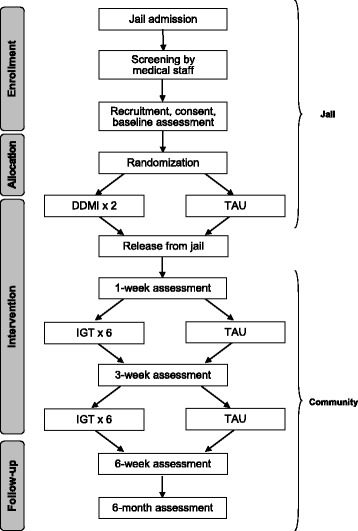
Pilot RCT study flowchart

This project was developed in partnership with multiple community organizations, including the county’s managed care organization, multiple community-based provider agencies, and the county jail. Each of these partners helped shape the proposal, including the development of the communication and research protocols and the selection of the treatments to be adapted. The Standard Protocol Items: Recommendations for Interventional Trials (SPIRIT) checklist is included as Additional file 1, and the SPIRIT figure for the RCT is presented in Fig. 1. The study flowchart is provided in Fig. 2.

### Interventions

Dual-diagnosis motivational interviewing (DDMI) [25–27] and integrated group therapy (IGT) [28–31] are two integrated dual disorder treatment (IDDT) programs with strong evidence bases. IDDT programs are the “standard of evidence-based treatment” for adults with CODs ([32], p. 317). After a review of multiple IDDT and non-IDDT treatment programs, our partner agencies believed DDMI and IGT to be appropriate candidates for adaptation to justice-involved populations.

#### Dual-diagnosis motivational interviewing

DDMI is an adaptation of motivational interviewing [26] that incorporates an integrated framework and accommodates the cognitive impairments and disordered thinking associated with CODs. For example, DDMI includes simplified open-ended questions, refined reflective listening skills, and integration of psychiatric issues into personalized feedback and decision making. Given its portability and effectiveness in few sessions [27], DDMI was thought to fit particularly well within the context of brief, but intensive jail-based treatment prior to community re-entry. In the current study, DDMI sessions lasting approximately 60 minutes will be delivered one on one to inmates in the jail prior to their first court appearance. When possible, inmates will receive a second DDMI session before anticipated release from jail.

#### Integrated group therapy

IGT is an evidence-based practice that has been cited by the National Institutes of Health (NIH) as one of five examples of “promising behavioral therapies” for adults with CODs ([33], p. 3). IGT uses cognitive and behavioral strategies to address substance use, psychiatric symptoms, and medication nonadherence via a focus on (1) promoting abstinence from drugs, including alcohol; (2) promoting adherence to psychiatric medications; (3) teaching mood- and thought-monitoring skills; (4) teaching social skills; and (5) improving other aspects of life functioning [30]. Because cognitive-behavioral therapies like IGT are effective for treating adults with CODs [34, 35], including the reduction of antisocial behaviors [36, 37], our partners believed that community-based IGT also was an appropriate treatment to adapt for the jail-to-community treatment continuum. The goal is to have inmates, within 1 week of release from jail, enroll in IGT and participate in 12 90-minute IGT sessions with four to eight other inmates for 6 weeks. IGT sessions will be run with an open enrollment format [30] to facilitate a quick jail-to-community transition period and rapid engagement in community-based treatment [9].

### Conceptual frameworks

Our treatment adaptation and implementation and communication protocol frameworks are described next.

#### Treatment adaptation framework

Our treatment adaptation framework is guided by the Risk-Need-Responsivity (RNR) model [38, 39] and the National Institute of Corrections’ (NIC’s) implementation competencies [40]. Within RNR, the risk principle indicates that individuals at highest risk of future adverse outcomes should be identified and resources allocated accordingly. The need principle asserts that interventions should target criminogenic needs related directly to adverse outcomes for the individual offender. The responsivity principle affirms that strategies should be sensitive to identified risk and needs, while being delivered in a way that considers individual factors that can affect treatment outcomes (e.g., learning style). There are eight NIC implementation competencies that also informed our treatment adaptation process: assess risk and need, enhance motivation, target interventions, cognitive-behavioral skill training, positive reinforcement, increase ongoing support, measure processes and practices, and measurement feedback.

Through the integration of RNR principles and NIC competencies, DDMI will be adapted for in-jail use with a focus on motivating the inmate for community re-entry, continuity of care, and uptake of routine outpatient services. IGT will be adapted for more intensive, staged delivery of sessions that target criminogenic risk and needs, while continuing to enhance motivation for uptake of routine outpatient services.

#### Implementation framework

Our implementation framework is guided by the Evidence-Based Interagency Implementation Model (EB-IIM) for community corrections [40]. This framework considers multiple, interacting levels at which implementation must occur, including the role of inner and outer settings in implementation. For example, we will focus on developing knowledge and building a foundation through the open trial (i.e., preparation phase), in addition to establishing agreed-upon expectations for the jail and community partners and aligning the jail’s policies and procedures to accommodate changes identified through the two trials. Finally, ongoing treatment adaptations, informed via open trial results, should improve the likelihood of sustaining a successful jail-to-community-based treatment continuum through and then beyond the pilot RCT.

#### Communication framework

Our communication protocols will be based on the Inter-organizational Relationship (IR) model that emphasizes situational factors, as well as process, structural, and outcome dimensions in inter- and intra-agency communication. The IR model defines the situational factors (e.g., need for resources) and process (e.g., intensity of information flow), structural (e.g., complexity), and outcome dimensions (i.e., perceived effectiveness) necessary for inter-agency collaboration. Thus, in addition to developing and testing inter-agency communication protocols (e.g., from jail to community treatment), we also will develop and test intra-agency communication protocols (e.g., how to manage scheduling conflicts between IGT and usual care services). The IR model has been used effectively across service settings [41–44] and maps onto the technical, procedural, and resource dimensions of the EB-IIM’s foundation core [40].

### Study design

The study consists of two phases: (1) an uncontrolled open trial and (2) a pilot RCT. During the open trial phase, we will collect data to inform the refinement of the research and communication protocols and the adapted interventions—broadly, the jail-to-community treatment continuum. For example, results from the uncontrolled open trial may result in changes to the inmate participant recruitment and consent process, procedures for facilitating inter-agency communication, and additional adaptations to the DDMI-IGT continuum training materials, manuals, or session handouts. All modifications to the research and communication protocols and to the adapted interventions will be made in collaboration with community partners throughout the uncontrolled open trial and implemented in the pilot RCT.

### Setting

The study will be conducted at a large urban county jail in the southeastern USA. The jail’s average daily census ranges between approximately 1300 and 1450 with one-quarter of those inmates identified as being in need of mental health evaluation beyond standard screening completed during booking. Within 24–48 hours, on average, all inmates have their first court appearance. From there, inmates are either (1) bonded out or have their charges dropped or (2) detained and sent to Misdemeanor or Felony Court. From Misdemeanor or Felony Court, inmates are released or sentenced.

### Inmate participants

Inmate participants will be broadly representative of adults with CODs in a large urban county jail. Twelve inmates will be enrolled in the *open trial* and 60 in the pilot *RCT*. Eligibility criteria are: 18 to 65 years of age; incarcerated in the county jail; a resident of the county; able to speak and read English; and meet Diagnostic and Statistical Manual of Mental Disorders, 4th edition (DSM-IV) diagnostic criteria for (1) drug or alcohol abuse or dependence and (2) a serious mental illness, including major depressive disorder, depressive disorder not otherwise specified (NOS), bipolar disorder I, II, or NOS, schizophrenia-spectrum disorder, schizoaffective disorder, schizophreniform disorder, brief psychotic disorder, delusional disorder, or psychotic disorder NOS, based on review of clinical records and input from available informants, including jail-based clinicians.

### Community partner participants

Community partner participants will be clinicians working in local behavioral healthcare agencies who will provide the intervention to treatment participants. Clinicians (*n* = 4) from a local behavioral health agency will be recruited and trained to provide the treatment to participants. Two clinicians will provide DDMI treatment to participants in the jail prior to release into the community. Two clinicians will provide the IGT treatment to participants in the community after release from jail. To be eligible, clinician participants must be providing clinical services to jail inmates with co-occurring substance use and mental health disorders, at least 18 years of age, able to speak/read English, and able to provide informed consent.

### Clinician training

Prior to the start of the open trial, we will provide 1 day of training on IDDT principles, frameworks for treatment adaptation and implementation, and the DDMI and IGT programs to all study clinicians. We also will provide a second day of training separately for the DDMI and IGT clinicians, focusing on jail- and community setting-specific issues relevant to each of the interventions. Clinicians will receive a booster training during the open trial that will address modifications and refinements to DDMI and IGT and the communication protocols, as informed by open trial data. Clinicians will be retrained prior to the start of the pilot RCT and will receive another booster session midway through the RCT.

### Participant identification and recruitment

Participants will be identified for potential participation in the study through routine screening measures at booking, including the Brief Jail Mental Health Screen [45] and other jail-specific mental health and substance use screening protocols. The Jail Mental Health Liaison will identify participants who meet the study inclusion criteria, which were noted above, and the jail-based clinician will approach potential participants (after booking but before the first court appearance) to inquire about interest in study participation.

#### Uncontrolled open trial

Recruitment and participation in the open trial will occur in two cycles to allow for evaluation and modification of the DDMI-IGT treatment continuum, as necessary. In the first cycle, six inmates will participate. The adapted interventions and communication and research protocols will be modified as necessary, based on inmate and clinician feedback. After modifications, another six inmates will participate in the second open trial cycle.

#### Pilot randomized controlled trial

For the pilot RCT, all inmates who consent to participate will be randomly assigned to one of two conditions: the DDMI-IGT treatment group or the control group. After consent, participants will receive their group assignment. All inmates will be given a preassigned study ID, each associated with assignment to either the treatment or control group, as determined by a random number generator. Participants will be informed of their allocation to study condition by the research interviewer. Allocation is not concealed, as participants will be asked to provide feedback on their satisfaction with, and preferences for, treatment (see Table 1).

**Table 1:** Uncontrolled open trial and pilot RCT measures and assessment schedule

					Assessment schedule					
Aim	Construct	Measure	Measure characteristics	Informant	OT	RCT				
						T0	T1	T2	T3	T4
1, 2.1, 2.2	Acceptability	Client Satisfaction Questionnaire-8 (CSQ-8) [49, 50]	8-item measure of satisfaction with DDMI-IGT treatment continuum	Inmates	X				X	
1, 2.1, 2.2	Acceptability	Narrative Evaluation of Intervention Interview (NEII) [51]	16-item interview for evaluating interventions	Inmates	X				X	
1, 2.1, 2.2	Acceptability, feasibility	Focus groups	Semi-structured groups about satisfaction with treatment continuum and protocols; implementation barriers, impact on jail and agencies	Community partners	X				X	
1	Fidelity	DDMI Adherence Form [52]	10-item measure of adherence and competence of DDMI sessions	Community partners	X					
1	Fidelity	IGT Adherence Form [28]	15-item measure of adherence and competence of IGT sessions	Community partners	X					
1, 2.1, 2.2	Feasibility	Exit interviews	Semi-structured interview of treatment success, barriers	Inmates	X				X	
1, 2.1, 2.2	Feasibility	Administrative records	Call logs; number of inter- and intra-agency contacts	Community partners	X				X	
1, 2.1, 2.2	Feasibility	Service Tracking Form	Treatment recruitment; sessions attended in jail, session attending in the community; graduation rates	Community partners	X				X	
2.3	Effectiveness: clinical	Addiction Severity Index (ASI) [53]	27-item measure of alcohol and substance use in prior 30 days, or since last assessment	Inmates		X	X	X	X	X
2.3	Effectiveness: clinical	Brief Psychiatric Rating Scale (BPRS) [71]	18-item measure of psychiatric symptom severity	Inmates		X	X	X	X	X
2.3	Effectiveness: clinical	Short Form Survey (SF-12) [72, 73]	12-item measure of health status and impairment	Inmates		X	X	X	X	X
2.3	Effectiveness: clinical	Epidemiological Catchment Area Interview [74]	14-item measure of self-reported service and medication use	Inmates		X	X	X	X	X
2.3	Effectiveness: clinical	Triarchic Psychopathy Measure [75]	20-item disinhibition scale of externalizing behaviors	Inmates		X				
2.3	Effectiveness: clinical	Heinrichs-Carpenter Quality of Life Scale, Plus [76]	31-item quality of life scale	Inmates		X	X	X	X	X
2.3	Effectiveness: public health	Criminal Justice Records	Electronic law enforcement records of arrest; jail tracking of criminal justice contacts	Community partners						X
2.3	Effectiveness: treatment preference	CODs treatment preferences [77, 78]	Visual analog ranking of preference for 8 aspects of treatment	Inmates					X	
—	Sample description	Demographic information	Diagnosis; age; race/ethnicity; sex	Community partners		X				

*OT* uncontrolled open trial, *RCT* randomized controlled trial, *T0* baseline assessment conducted in jail at treatment program enrollment, *T1* within 1 week of jail discharge, *T2* 3 weeks after jail discharge (mid-IGT treatment), *T3* 6 weeks after jail discharge (end of IGT treatment), *T4* 6 months after jail discharge

#### Treatment group

Inmates assigned to the treatment group will participate in the DDMI-IGT continuum. Between four and eight groups of participants will complete the DDMI-IGT treatment during the pilot RCT, depending on how many participants are enrolled in each IGT cycle. No more than 8 inmates will participate in IGT at any given time. In addition to the DDMI-IGT treatment continuum, inmates in the treatment condition also will receive any and all necessary usual care across jail and community settings.

#### Control group

Inmates assigned to the control condition will receive treatment as usual (TAU) and will not be enrolled in the DDMI-IGT treatment continuum. That is, inmates in the control group will continue to receive any and all usual care services across jail and community settings, as do any inmates regardless of participation in this study.

As noted, participants across both conditions will receive any and all clinically indicated usual care services across the jail and community sites. The delivery of those services, including their discontinuation or modification if needed, will be based on the treating clinician’s judgement and appropriate treatment planning within the context of the jail and community-based treatment system supporting the implementation of this project.

### Assessment schedule and measures

#### Open trial

To address Aim 1, we will collect qualitative and quantitative data regarding acceptability of, fidelity to, and feasibility of the treatment from inmates and clinicians during the uncontrolled open trial (see Table 1). *Acceptability* will be evaluated to determine participant and clinician satisfaction with the communication protocols and the DDMI-IGT treatment continuum, using the Client Satisfaction Questionnaire-8 (CSQ-8) [46, 47], Narrative Evaluation of Intervention Interview (NEII) [48], semi-structured interviews, focus groups, and administrative records. *Fidelity* will be examined at the end of the open trial to determine the degree to which clinicians adhered to the adapted treatment programs based on a review of audio recorded treatment sessions and DDMI (Moyers, Martin, Manuel, Miller, Ernst. Revised global scales:motivational interviewing treatment integrity 3.1. 1 (MITI 3.1. 1). Unpublished.) and IGT [28] Adherence Forms. *Feasibility* will be evaluated to determine the success of treatment implementation, enrollment, and coordination, as well as the feasibility of research and communication protocols, using data collected from semi-structured interviews, focus groups, and administrative records.

#### Pilot randomized controlled trial

To address Aim 2, we will collect inmate and community partner outcome data to evaluate treatment effectiveness, feasibility, and acceptability of the pilot RCT (see Table 1). Treatment *effectiveness* will be assessed using three categories of outcome variables: (1) clinical, including inmate substance use, psychiatric symptoms, health status, service use, and quality of life; (2) criminal justice, including contacts and re-arrest, which will serve as the primary outcomes of the pilot RCT; and (3) treatment preferences. These outcomes will be assessed five times, including an in-jail baseline assessment and four additional community-based assessments spanning 1 week to 5 months after jail discharge (see Table 1). In addition to these effectiveness outcomes, we additionally will measure implementation outcomes related to the interventions and protocols. Specifically, *feasibility* and *acceptability* will be assessed using the same measures from the open trial, including inmate exit interviews, community partner focus groups, and administrative record review. The primary explanatory variable will be the experimental condition; that is, whether participants were assigned to either the treatment group (DDMI-IGT + TAU) or the control group (TAU only). Additionally, we will collect sociodemographic data for participants, including diagnosis, race/ethnicity, age, and sex, obtained through clinical and administrative records. Inclusion of these variables will allow examination of potential differences in treatment outcome as a function of each variable while controlling for sociodemographic factors in statistical analyses.

### Analytic plan and statistical methods

#### Aim 1

We will conduct Aim 1 analyses to evaluate qualitative and quantitative data regarding the feasibility of, fidelity to, and acceptability of the DDMI-IGT continuum and protocols. All transcribed qualitative data from interviews and focus groups will be coded according to coding guidelines which will concentrate on deductive themes focusing on the feasibility and acceptability of treatment programs and protocols. A subset of transcripts will be coded independently by two team members in Atlas.ti using iterative content analysis [49]. Discrepancies will be resolved by refining codes and definitions through team discussion, until >90% agreement is reached. After all transcripts are coded, data will be searched and data output analyzed to build an understanding of feasibility and acceptability and possible revisions. For example, potential broad category codes could include “feasibility challenges,” “feasibility facilitators,” and “feasibility recommendations” with lower level codes indicating data collected from providers and participants. Low-level codes will be grouped into broader categories when possible. Additionally, we will calculate and review descriptive statistics of administrative data (e.g., call logs, frequency of inter- and intra- communication, average number of treatment sessions attended) to assess treatment and protocol feasibility. Findings will be used to refine treatment adaptations and protocols as needed prior to conducting the pilot RCT.

#### Aim 2

We will examine baseline clinical, legal, and demographic differences between groups to determine if RCT treatment group randomization was successful [50]. Additionally, we will assess demographic differences between consenters and refusers, reasons for refusal, and retention rates.

To address Aims 2.1 and 2.2 regarding the feasibility and acceptability of and fidelity to the treatment and protocols, we will integrate qualitative and quantitative data by transforming qualitative data (e.g., noting the occurrence of themes in interviews and focus groups) to support or refute quantitative results (i.e., data merging) [51–53]. All qualitative data collected from inmates, clinicians, and jail personnel will be analyzed using the coding approach described above for Aim 1. Feasibility will be evidenced by the acceptable rates of participant exposure (85% complete DDMI; attend >1 IGT session/week) and retention (>75% completion rate) in the treatment continuum [54–56]. Acceptability to clinicians will be evidenced by focus group and interview themes reflecting an overall more positive than negative perception (e.g., endorsement for continuing the program, even if minor inconveniences are noted). Acceptability to inmates will be evidenced by average CSQ-8 item scores >3 [57] and interview themes reflecting more positive than negative perceptions. Fidelity will be evidenced by mean item scores >4 (Moyers, Martin, Manuel, Miller, Ernst. Revised global scales:motivational interviewing treatment integrity 3.1. 1 (MITI 3.1. 1). Unpublished) for the DDMI and >3 [28] for the IGT fidelity ratings.

We will use a generalized linear mixed model (GLMM) to address Aim 2.3 regarding the effectiveness of the DDMI-IGT treatment condition. In addition to inferential tests of significance and associated confidence intervals, effect sizes will be estimated. Our approach will be extended to estimate standardized differences between the means for planned contrasts in the amount of change that has taken place between pairs of times. Effect sizes will be ranked from highest to lowest, tabled, and graphed. Mindful of concerns regarding estimation of effect sizes in pilot studies and the accuracy of estimates of replication, our goal will be to identify a consistent pattern in the results that will help determine the choice of primary outcome measures in future research [58, 59].

Although not a primary aim of this study, an important clinical question is the extent to which the intervention might be differentially effective as a function of inmate characteristics at treatment entry. We believe four factors might moderate intervention effectiveness and thus merit attention in our future research: diagnosis or psychiatric severity [8, 60]; substance use, including differential use of alcohol or drugs [8, 61], psychiatric symptoms [61–63]; and criminal [62, 63] or antisocial history, including disinhibition [64]. Following our GLMM analyses, we will undertake exploratory supplemental analyses by fitting expanded statistical models that include these variables as additional main effects and, more importantly, as interactions with treatment condition, to assess the extent to which each might moderate the treatment condition effect.

### Monitoring

A Data Safety and Monitoring Board (DSMB), whose members will be independent of the funding agency, the National Institute on Drug Abuse (NIDA), will be brought together to oversee the study’s activities and to ensure the safety of human subjects, validity of findings, and need for further data collection. The study principal investigator (PI), Dr. Van Dorn, will interact with the DSMB at the DSMB’s discretion (at least two meetings per year), providing them with material to review, monitor, evaluate, audit, and make recommendations regarding: (1) protocols, informed consent procedures, and safety plans; (2) study progress (i.e., recruitment and retention, risk/benefit ratio for subjects, adherence to timetable, quality of data); (3) the impact of new treatment developments on the risk/benefit ratio of the study; (4) continuation, modification, or termination of ongoing studies based on adverse events or beneficial outcomes; (5) interim analyses; (6) confidentiality of trial data and results of monitoring; and (7) procedures likely to increase subjects’ burden, to raise ethical concerns, or to give the appearance of a conflict of interest.

The PI will make the following available to the DSMB: (1) all adverse events (RTI International protocol requires the reporting, in writing, of all adverse events within 5 days of the study team becoming aware of the adverse event. All adverse event forms will be made available to the DSMB as well as tables summarizing the occurrence of specific events.); (2) all interim data analyses; (3) analyses requested by the DSMB; and (4) all reports to NIDA and all publications. Finally, data management procedures, including those for data entry and quality checks, coding, security, and storage are available from the study PI.

### Power analyses

The proposed study will have a small, but appropriate [65] sample size of 60. This study is developmental in nature, which informs how we have chosen to address the choice of error rates and power to detect effects in our clinical and public health outcomes. Prior research suggests small to medium substance use and psychiatric symptom effects of DDMI [25, 27] and IGT [28–30]. Therefore, type I error rate α = 0.15 was chosen so as not to limit power to conduct inferential analyses, giving an 80% chance (1 – β = .80) to detect a difference between the treatment conditions that would explain 9% of the total variance [θ^2^ = .09] in an outcome variable if the null hypothesis were false (calculated using G*Power 3.1 [66]). This approach allows for increased opportunity to detect a smaller effect, while running the risk that the effect will not replicate. This course of action is appropriate for a developmental study and is consistent with prior research involving adults with SMI and preliminary-stage multivariable models [61].

## Discussion

Adults with mental illness suffer disproportionately from drug and alcohol problems and are overrepresented in jails. Our study aims to address the problem of limited treatment for this population. Therefore, we will adapt DDMI and IGT for jail inmates with CODs and evaluate the acceptability, feasibility, fidelity, and effectiveness of implementing the adapted treatment continuum and associated research and communication protocols.

### Innovation

Our study will advance current research and practice regarding evidence-based treatment for inmates with CODs and associated research methodologies. We will adapt two interventions, DDMI and IGT, for justice-involved adults with CODs. Our project is grounded in evidence-based conceptual and treatment processes and will have the potential to improve substance use, psychiatric, arrest, and routine outpatient treatment uptake outcomes among an underserved and high-risk population. This type of research is needed to address the increasing rates of persons with CODs in jail populations and associated treatment failure. Second, the development and implementation of RCT methodologies is needed within and across jail and community settings to demonstrate the feasibility, acceptability, and effectiveness of adapting treatments and conducting RCTs with this population. Accordingly, this study may influence the progression of subsequent CODs and jail research and encourage others to adopt similar rigorous and system-bridging approaches.

### Limitations

Our study has several limitations in the design and implementation. Although court orders can be successful in increasing treatment participation among adults with CODs, substance use disorders (SUDs), and SMIs [67–69], we do not propose any judicial oversight. This decision is in line with the preferences of our community partners and other ongoing studies [70] and has proven to be successful in retaining participants. Given our focus on one jail, the generalizability of our findings may be limited; however, generalizability of our findings can be examined in a future full-scale RCT. Allowing access to TAU in both experimental conditions also presents several potential limitations. As much as possible, our community agencies will have different clinicians deliver the DDMI-IGT and usual care services to experimental and control participants; however, DDMI-IGT and usual care will be provided in the same agencies, which may result in treatment contamination. Fidelity assessments will allow us to monitor DDMI-IGT treatment integrity and to address this potential contamination. It also is likely that, across both experimental conditions, inmates will receive different types and intensities of usual care services, resulting in treatment heterogeneity. This will not affect study interpretability, however, because service intensity will be titrated to individual client needs, consistent with the RNR framework [39]. If our DDMI-IGT continuum increases the amount or duration of usual care service uptake, the extent to which improvements in outcomes can be “explained” by amount or duration of usual care services could be explored by statistically controlling for these services and comparing the treatment conditions, e.g., through analysis of covariance (ANCOVA) models. Lastly, given that our study is focused on the preliminary treatment adaptations and feasibility, we have not focused on differential effects of DDMI and IGT within our treatment continuum.

### Trial status

Participant recruitment for the RCT is ongoing.

## Additional file

Additional file 1: SPIRIT checklist. Items addressed in the current clinical trial. (DOC 123 kb)

## Abbreviations

BJMHS: Brief Jail Mental Health Screen
CODs: Co-occurring mental and substance use disorders
DDMI: Dual-diagnosis motivational interviewing
EB-IIM: Evidence-Based Interagency Implementation Model
IDDT: Integrated dual disorder treatment
IGT: Integrated group therapy
NIC: National Institute of Corrections
NOS: Not otherwise specified
RCT: Randomized controlled trial
RNR: Risk-Need-Responsivity
SMI: Serious mental illness
TAU: Treatment as usual.

## References

[1] Steadman HJ (2009). Prevalence of serious mental illness among jail inmates. Psychiatric Services.

[2] Drake RE, Brunette MF, Galanter M (1998). Complications of severe mental illness related to alcohol and drug use disorders. Recent developments in alcoholism.

[3] Van Dorn RA (2012). Assessing illicit drug use among adults with schizophrenia. Psychiatry Res.

[4] Desmarais SL, et al. Accuracy of self-report, biological tests, collateral reports and clinician ratings in identifying substance use disorders among adults with schizophrenia. Psychology Addictive Behav. 2013;27(3):774-87. 10.1037/a0031256 PMC3654003 23276310

[5] Dickey B, Azeni H (1996). Persons with dual diagnoses of substance abuse and major mental illness: their excess costs of psychiatric care. Am J Public Health.

[6] Essock SM (2006). Comparison of ACT and standard case management for delivering integrated treatment for co-occurring disorders. Psychiatric Services.

[7] Friedmann PD (2012). Collaborative behavioral management among parolees: drug use, crime and re‐arrest in the Step'n Out randomized trial. Addiction.

[8] Van Dorn R, Volavka J, Johnson N (2012). Mental disorder and violence: is there a relationship beyond substance use?. Soc Psychiatry Psychiatric Epidemiology.

[9] Van Dorn RA (2013). Effects of outpatient treatment on risk of arrest of adults with serious mental illness and associated costs. Psychiatric Services.

[10] Drake RE (2001). Implementing dual diagnosis services for clients with severe mental illness. Psychiatric Services.

[11] Ford L, Snowden LR, Walser EJ (1991). Outpatient mental health and the dual-diagnosis patient: utilization of services and community adjustment. Evaluation Progr Plan.

[12] Chandler RK (2004). Challenges in implementing evidence‐based treatment practices for co‐occurring disorders in the criminal justice system. Behav Sci Law.

[13] Drake RE (2002). Psychosocial aspects of substance abuse by clients with severe mental illness. J Nerv Mental Dis.

[14] Drake RE (1998). Review of integrated mental health and substance abuse treatment for patients with dual disorders. Schizophrenia Bull.

[15] Drake RE, Mueser KT (2000). Psychosocial approaches to dual diagnosis. Schizophrenia Bull.

[16] Mueser KT (2002). Illness management and recovery: a review of the research. Psychiatric Services.

[17] Cohen F, Dvoskin J (1992). Inmates with mental disorders: a guide to law and practice. Mental Physical Disability Law Report.

[18] Osher F, Steadman HJ, Barr H (2003). A best practice approach to community reentry from jails for inmates with co-occurring disorders: the APIC model. Crime and Delinquency.

[19] Munetz MR, Griffin PA (2006). Use of the sequential intercept model as an approach to decriminalization of people with serious mental illness. Psychiatric Services.

[20] Haggerty JL (2003). Continuity of care: a multidisciplinary review. BMJ.

[21] Adair CE (2003). History and measurement of continuity of care in mental health services and evidence of its role in outcomes. Psychiatric Services.

[22] Crawford MJ (2004). Providing continuity of care for people with severe mental illness. Soc Psychiatry Psychiatric Epidemiology.

[23] Kuno E (2005). A service system planning model for individuals with serious mental illness. Mental Health Services Res.

[24] Swartz MS, Swanson JW (2004). Involuntary outpatient commitment, community treatment orders, and assisted outpatient treatment: what's in the data?. Canad J Psychiatry.

[25] Martino S (2006). A randomized controlled pilot study of motivational interviewing for patients with psychotic and drug use disorders. Addiction.

[26] Martino S (2002). Dual diagnosis motivational interviewing: a modification of motivational interviewing for substance-abusing patients with psychotic disorders. J Subst Abuse Treat.

[27] Martino S (2000). Motivational interviewing with psychiatrically ill substance abusing patients. Am J Addictions.

[28] Weiss R (2007). A randomized trial of integrated group therapy versus group drug counseling for patients with bipolar disorder and substance dependence. Am J Psychiatry.

[29] Weiss RD (2000). Group therapy for patients with bipolar disorder and substance dependence: results of a pilot study. J Clinical Psychiatry.

[30] Weiss RD, Connery HS (2011). Integrated group therapy for bipolar disorder and substance abuse.

[31] Weiss RD (2009). A “community-friendly” version of integrated group therapy for patients with bipolar disorder and substance dependence: a randomized controlled trial. Drug Alcohol Dependence.

[32] Ziedonis DM, et al. Improving the care of individuals with schizophrenia and substance use disorders: consensus recommendations. J Psychiatric Practice. 2005;11(5):315-39. 10.1097/00131746-200509000-00005 PMC2599914 16184072

[33] National Institute on Drug Abuse (2008). Comorbidity: addiction and other mental illnesses.

[34] Monti PM (2002). Treating alcohol dependence: a coping skills training guide.

[35] Beck AT (2001). Cognitive therapy of substance abuse.

[36] Butler AC (2006). The empirical status of cognitive-behavioral therapy: a review of meta-analyses. Clinical Psychology Rev.

[37] Landenberger NA, Lipsey MW (2005). The positive effects of cognitive–behavioral programs for offenders: a meta-analysis of factors associated with effective treatment. J Experimental Criminology.

[38] Taxman FS, Thanner M, Weisburd D (2006). Risk, need, and responsivity (RNR): it all depends. Crime Delinquency.

[39] Bonta J, Andrews DA (2007). Risk-need-responsivity model for offender assessment and rehabilitation.

[40] Taxman FS, Belenko SR (2012). Implementing evidence-based practices in community corrections and addiction treatment.

[41] Delaney FG (1994). Muddling through the middle ground: theoretical concerns in intersectoral collaboration and health promotion. Health Promotion Internat.

[42] Beatrice DF (1991). Inter-agency coordination: a practitioner's guide to a strategy for effective social policy. Administration Soc Work.

[43] Fottler MD (1982). Multi-institutional arrangements in health care: review, analysis, and a proposal for future research. Academy of Manag Rev.

[44] Fox J, Merwin E, Blank M (1995). De facto mental health services in the rural south. J Health Care for the Poor and Underserved.

[45] Steadman HJ (2005). Validation of the Brief Jail Mental Health Screen. Psychiatric Services.

[46] Atkisson C, Greenfield T, Maruish M (1994). The Client Satisfaction Questionnaire-8 and the Service Satisfaction Questionnaire-30, psychological testing: treatment planning and outcome assessment.

[47] Nguyen TD, Attkisson CC, Stegner BL (1983). Assessment of patient satisfaction: development and refinement of a service evaluation questionnaire. Evaluation Progr Plan.

[48] Hasson-Ohayon I, Roe D, Kravetz S (2006). A qualitative approach to the evaluation of psychosocial interventions for persons with severe mental illness. Psychological Services.

[49] Weber RP (1990). Basic content analysis.

[50] Rosen A, Proctor EK (1981). Distinctions between treatment outcomes and their implications for treatment evaluation. J Consulting Clinical Psychology.

[51] Creswell JW, Plano Clark VL (2011). Designing and conducting mixed methods research.

[52] Creswell JW (2011). Best practices for mixed methods research in the health sciences.

[53] Sandelowski M, Voils CI, Knafl G (2009). On quantitizing. J Mixed Methods Res.

[54] Carroll KM (2006). Motivational interviewing to improve treatment engagement and outcome in individuals seeking treatment for substance abuse: a multisite effectiveness study. Drug Alcohol Dependence.

[55] Baekeland F, Lundwall L (1975). Dropping out of treatment: a critical review. Psychological Bull.

[56] O’Brien A, Fahmy R, Singh SP (2009). Disengagement from mental health services. Soc Psychiatry Psychiatric Epidemiology.

[57] Wise EA (2010). Evidence-based effectiveness of a private practice intensive outpatient program with dual diagnosis patients. J Dual Diagnosis.

[58] Kraemer HC (2006). Caution regarding the use of pilot studies to guide power calculations for study proposals. Archives Gen Psychiatry.

[59] Miller J, Schwarz W (2011). Aggregate and individual replication probability within an explicit model of the research process. Psychological Methods.

[60] Van Dorn RA (2011). Risk of arrest in persons with schizophrenia and bipolar disorder in a Florida Medicaid program: the role of atypical antipsychotics, conventional neuroleptics, and routine outpatient behavioral health services. J Clinical Psychiatry.

[61] Swanson JW (2006). A national study of violent behavior in persons with schizophrenia. Archives General Psychiatry.

[62] Swanson JW (2008). Alternative pathways to violence in persons with schizophrenia: the role of childhood antisocial behavior problems. Law Human Behav.

[63] Swanson JW (2008). Comparison of antipsychotic medication effects on reducing violence in people with schizophrenia. Br J Psychiatry.

[64] Ball SA (2005). Personality traits, problems, and disorders: clinical applications to substance use disorders. J Res Personality.

[65] Proctor EK (2012). Writing implementation research grant proposals: ten key ingredients. Implement Sci.

[66] Faul F (2007). G* Power 3: a flexible statistical power analysis program for the social, behavioral, and biomedical sciences. Behav Res Methods.

[67] Swartz MS (2010). Assessing outcomes for consumers in New York's assisted outpatient treatment program. Psychiatric Services.

[68] Gilbert AR (2010). Reductions in arrest under assisted outpatient treatment in New York. Psychiatric Services.

[69] Van Dorn RA (2010). Continuing medication and hospitalization outcomes after assisted outpatient treatment in New York. Psychiatric Services.

[70] Desmarais SL (2012). Characteristics of START assessments completed in mental health jail diversion programs. Behav Sci Law.

[71] Overall JE, Gorham DR (1962). The Brief Psychiatric Rating Scale. Psychol Rep.

[72] Gandek B, Ware JE, Aaronson NK, Apolone G, Bjorner JB, Brazier JE, Bullinger M, Kaasa S, Leplege A, Prieto L, Sullivan M (1998). Cross-Validation of Item Selection and Scoring for the SF-12 Health Survey in Nine Countries. J Clin Epidemiol.

[73] McHorney CA, Ware Jr JE, Raczek AE. The MOS 36-Item Short-Form Health Survey (SF-36): II. Psychometric and clinical tests of validity in measuring physical and mental health constructs. Med Care. 1993:247–263. 10.1097/00005650-199303000-00006 8450681

[74] Blazer D, George LK, Landerman R, Pennybacker M, Melville M, Woodbury M, Manton KG, Jordan K (1985). Psychiatric Disorders. Arch Gen Psychiatry.

[75] Patrick CJ, Fowles DC, Krueger RF (2009). Triarchic conceptualization of psychopathy: Developmental origins of disinhibition, boldness, and meanness. Dev Psychopathol.

[76] Heinrichs DW, Hanlon TE, Carpenter WT (1984). The Quality of Life Scale: An Instrument for Rating the Schizophrenic Deficit Syndrome. Schizophr Bull.

[77] Swartz MS, Swanson JW, Van Dorn RA, Elbogen EB, Shumway M (2006). Patient Preferences for Psychiatric Advance Directives. Int J Forensic Ment Health.

[78] Swartz MS, Swanson JW, Wagner HR, Hannon MJ, Burns BJ, Shumway M (2003). Assessment of four stakeholder groups’ preferences concerning outpatient commitment for persons with schizophrenia. Am J Psychiatry.

